# Genetic basis of voluntary water consumption in two divergently selected strains of inbred mice

**DOI:** 10.1002/vms3.192

**Published:** 2019-08-02

**Authors:** Maria Haag, Kevin Wells, William Lamberson

**Affiliations:** ^1^ Division of Animal Sciences University of Missouri Columbia Missouri

**Keywords:** genetics, mice, water consumption

## Abstract

**Background:**

Inbred mouse strains with normal renal function show a substantial difference in daily water consumption across strains. This study uses two strains of inbred mice C57BR/CDJ (BR), which are high consumers, and C57BL/10J (BL), which are low consumers, their reciprocal *F*_1_ crosses, inter se bred *F*_2_s and backcrosses produced by breeding high consuming *F*_2_ animals to the low consumer parent strain and low consuming *F*_2_ animals to the high consuming parent strain. Consumption was corrected for body weight prior to analysis.

**Methods:**

The effective number of genes controlling water consumption was estimated using the Castle–Wright estimator. Additive and dominance genotypic values as well as the degree of dominance were calculated using estimated strain means.

**Results:**

According to Castle–Wright, a minimum of 10 factors were estimated to affect the difference in consumption across the two strains. Between seven and eight are expected to be high effect factors. Using the Zeng adjustment, it was determined that 30–40 factors potentially affect the difference in consumption.

**Conclusions:**

These numbers were surprising but may be related to several sources of variation present in the BR strain. A negative degree of dominance indicated the BL strain has more dominant factors.

## 1 INTRODUCTION

It is estimated that by 2025 more than two thirds of the world's population will be living in a water‐scarce environment (UN‐Water, 2013). The issue is intensified by increased demands for water due to a growing population, changing diets and development (de Fraiture & Wichelns, 2010; Godfray et al., 2016; Schlink, Nguyen, & Viljoen, 2010). This shortage demands innovative techniques for reducing water usage. While direct consumption of water by livestock only accounts for about 1% of water usage (Maupin et al., 2010) there is certainly merit in its reduction. This is particularly true in developing countries, such as those in northern Africa, where water is scare and demand for animal products is on the rise (Allan, 2001). These areas need animals that can consume limited amounts of water whilst obtaining maximal growth for meat production. There is also a need for animals to perform draught work as crop production increases in developing countries (Schlink et al., 2010). Again, animals that are less affected by water scarcity could be beneficial.

Many animals already show adaptations that make them more suitable to these challenging environments. In fact, adaptations of “tropical” cattle are very well characterized. However, the genetic controls of many of these adaptations are unknown (Barendse, 2017). Under the premise of genetic control being conserved across species, a long‐term backcross study to isolate genes that control water consumption in two divergently selected strains of mice was designed.

In standard laboratory conditions, inbred mouse strains with unaltered kidney function show about a fourfold range of daily water consumption (Tordoff, Bachmanov, & Reed, 2007). Using this information, a high consuming strain and a low consuming strain were selected. The objective of the present study is to estimate the effective gene number controlling the difference in consumption between these two strains and estimate additive and genotypic values for the trait.

## 2 METHODS

### 2.1 Experimental animals

Foundation C57BL/10J (Black) and C57BR/CDJ (Brown) were purchased from Jackson Laboratories (Bar Harbor, ME). These strains were chosen based on previously quantified difference (Blacks: 0.35 mL/g^0.667^/d; Browns: 0.80 mL/g^0.667^/d) in water consumption (Bachmanov, et al., 2002; Tordoff et al., 2007). The strains show no difference in renal function (Thaisz et al., 2012). These animals were bred to establish two single‐strain colonies at the University of Missouri, Columbia. In accordance with the suggested design for diallel crosses (Griffing, 1956), females from each strain were bred to males from the opposite to produce *F*_1_ reciprocal crosses. *F*_1_ animals were then bred *inter se* to produce the *F*_2_ generation. To produce backcross animals, the two highest consuming and two lowest consuming male, *F*_2_ animals were selected for breeding. Animals were then bred to the opposite parental strain; high consumers to Blacks and low consumers to Browns. Backcrosses produced from the high consumer were referred to as High Backcross 1s (HB1s). Those from the low consumer were referred to as Low Backcross 1s (LB1s).

Consumption and weight data were collected on 848 animals: 68 Black, 81 Brown, 117 *F*_1_, 338 *F*_2_, 129 HB1 and 115 LB1. Animals were housed in plastic tub containers with corn cob bedding per ACUC approved protocol 8565. All males were individually housed, breeder females were group housed and experimental animals were individually housed during water consumption measurements. The temperature was maintained at 24 ± 1°C.

### 2.2 Weight and consumption measurement

Animals were weaned at 4 weeks, weighed, and separated into individual cages with custom‐built 25‐ml serological pipette water bottles based upon a previous design (Bachmanov, Tordoff, & Beauchamp, 1996). To see a more specific description of data collection methods, refer to Haag, Wells, and Lamberson (2018).

### 2.3 Statistical analysis and selection

Regression analysis of measured water intake on body weight, strain and sex was conducted prior to other analyses.

To estimate means, variances and standard errors mixed model analysis of adjusted consumption were conducted using PROC MIXED in SAS software. Adjusted consumption was designated as the dependent variables and fitted to a linear mixed model in the analysis:

$$N_{\mathrm{ijkl}}=\mu +{\text{strain}}_{i}+{\text{sex}}_{j}+{\text{sire}}_{k}\left({\text{strain}}_{l}\right)+{\text{strain}}_{i}*{\text{sex}}_{j}+e_{\mathrm{ijkl}}$$

In this model, *N*_*ijkl*_ is the dependent variable, adjusted consumption, *μ* is the mean, strain_*i*_, sex_*j*_ and strain_i_*sex_*j*_ are the designated fixed effects for strain, sex and the strain*sex interaction, respectively, sire_*k*_ (strain)_*i*_ is the sire within strain random effect and, finally, *e*_*ijkl*_ is the error term.

Effective gene number was estimated using the Castle–Wright estimator (Castle, 1921; Cockerham, 1986; Lande, 1981; Wright, 1968):

$$n_{\text{e}}=\frac{{\left(\overset{¯}{z}\left(P_{1}\right)-z\left(P_{2}\right)\right)}^{2}-\text{Var}\left[\overset{¯}{z}(P_{1})\right]-\text{Var}\left[\overset{¯}{z}\left(P_{2}\right)\right]}{8*\sigma_{S}^{2}}$$

In this equation, *n*_e_ is the estimated number of factors affecting a trait of interest, *zP*_1_ and *zP*_2_ are the observed means of the trait for each parental strain, Var[*zP*_1_] and Var[*zP*_2_] are the sampling variances for each parental strain estimated by squaring observed standard errors and $\sigma_{S}^{2}$ is the estimated segregation variance for each trait. Segregation variance estimation is described later in this section. The number of effective factors was estimated using the square root of the variance of *n*_e_.

To yield a less biased estimation, Zeng's adjustment equation (Zeng, 1992) was used to calculate *n*_e_:

$$n=\frac{2\overset{¯}{c}n_{\text{e}}+C_{\alpha }\left(n_{\text{e}}-1\right)}{\left[1-n_{\text{e}}\left(1-2\overset{¯}{c}\right)\right]}$$

To evaluate assumptions associated with the Castle–Wright estimator, adjusted consumption data were evaluated for epistasis and additivity. Epistasis testing was completed using the equation (Lynch & Walsh, 1998):

$$\Delta =z\left(F_{2}\right)-\left(\frac{z\left(P_{1}\right)+z\left(P_{2}\right)}{4}+\frac{z(F_{1})}{2}\right)$$

where Δ represents the epistatic estimate, *z*(*F*_2_), *z*(*P*_1_), *z*(*P*_2_) and *z*(*F*_1_) represent the observed line means for the *F*_2_, Black, Brown and *F*_1_ animals, respectively. As the observed Δ was not zero for the dataset, a sampling variance of Δ was estimated using the following equation:

$$\text{Var}\left(\Delta \right)=\text{Var}\left[z\left(F_{2}\right)\right]+\frac{Var[z\left(F_{1}\right)]}{4}+\frac{Var\left[z\left(P_{1}\right)\right]+Var\left[z\left(P_{2}\right)\right]}{16}$$

where Var(Δ) is the sample variance for estimated Δ value, Var[*z*(*F*_2_)], Var[*z*(*F*_1_)], Var[*z*(*P*_1_)] and Var[*z*(*P*_2_)] are the observed sampling variances for *F*_2_, *F*_1_, Black and Brown animals, respectively. The ratio of |Δ|/$\sqrt{\text{Var}(\Delta )}$ then provides a *t* test for evaluation of significance.

The joint scaling test (Cavalli, 1952; Gale, Mather, & Jinks, 1977; Mather & Jinks, 1971) was used to evaluate additivity. This test was designed to evaluate the increased variance observed in the *F*_2_ generation. The test begins by fitting data to the simplest, additive model:

$$M=\left[\begin{array}{cc} 1 & 1\\ 1 & -1\\ 1 & 0\\ 1 & 0\\ 1 & 0.5\\ 1 & -0.5 \end{array}\right]$$

Matrix *M* represents the coefficients of effects for *μ* and *α*_c_ for Brown, Black, *F*_1_, *F*_2_, HB1 and LB1, respectively. A chi‐squared test ultimately determines the adequacy of the model for the data.

There are several methods to estimate segregation variance; however, these methods can produce highly variable results (Lande, 1981; Lynch & Walsh, 1998). To avoid high variability, the least squares analysis method (Lynch & Walsh, 1998) was selected:

$$M=\left[\begin{array}{ccc} 1 & 0 & 0\\ 0 & 1 & 0\\ 0.5 & 0.5 & 0\\ 0.5 & 0.5 & 1\\ 0.75 & 0.25 & 0.5\\ 0.25 & 0.75 & 0.5 \end{array}\right]$$

The matrix *M* contains the coefficients of the variance components for Black, Brown, *F*_1_, *F*_2_, HB1 and HB2, respectively. Iterative analysis eventually results in the final least squares parameter estimates for *σ* ^2^(*P*_1_), *σ* ^2^(*P*_2_) and $\sigma_{S}^{2}$.

Additive and dominance genotypic values were calculated in the population with means from Black, Brown and *F*_1_ animals (Falconer & Mackay, 1996). Additive genotypic value (*a*), was determined using the equation:

$$a=\frac{\mu_{P_{2}}-\mu_{P_{1}}}{2}$$

In this equation, the parental line with the highest phenotypic value should be first in the numerator, or Black from Brown. Dominance genotypic value (*d*) was determined using the equation:

$$d=\mu_{F_{1}}-\frac{\mu_{P_{1}}+\mu_{P_{2}}}{2}$$

In this equation, the mean of the parental strains is subtracted from the mean of the *F*_1_ strain. To estimate the degree of dominance, the dominance value was divided by the additive value.

## 3 RESULTS

A significant sex*strain interaction was observed in the dataset. This previously observed interaction (Haag et al., 2018; McGivern, Henschel, Hutcheson, & Pangburn, 1996; Reed, Bachmanov, & Tordoff, 2007), was accounted for by analysing each sex separately for all analyses in the study.

Brown animals consumed more water (*P* < .0001) than Black animals (Table 1). *F*_1_ animals had higher water consumption but were much closer to that of the Black animals (Table 1). *F*_2_ animals showed a range of phenotypes encompassing both parental phenotypes as well as higher variance than the *F*_1_ animals. However, variance in the *F*_2_ was not as high as that observed in the Brown animals. Backcross animals showed means and variances moving towards parental strain values each generation.

**Table 1:** Number of animals per sex and strain, least squares means + *SE* (ml/g/wt^0.67^) of water consumption for each sex and strain and the estimated variance for each sex and strain

Line	*N*	*μ* ± *SE*	$\sigma_{S}^{2}$
Black Female	40	0.619 ± 0.039	0.0072
Black Male	28	0.582 ± 0.042	0.0086
Brown Female	53	1.459 ± 0.050	0.0805
Brown Male	28	1.136 ± 0.044	0.0651
*F*_1_ Female	56	1.034 ± 0.022	0.0193
*F*_1_ Male	61	0.874 ± 0.022	0.0103
*F*_2_ Female	179	0.964 ± 0.012	0.0289
*F*_2_ Male	159	0.876 ± 0.013	0.0157
HB1 Female	72	0.741 ± 0.032	0.0202
HB1 Male	57	0.690 ± 0.032	0.0111
LB1 Female	64	0.934 ± 0.031	0.0300
LB1 Male	51	0.924 ± 0.033	0.0200

Prior to factor number estimation, the epistatic, additive and dominance effects were analysed to determine how well the data fit the assumptions for Castle–Wright. Both sexes showed a significant indication (*P* < .0001) of epistatic effects (Table 2), a violation of assumptions that would minimize the number of factors identified. Both sexes were, however, adequately fit by the additive model as evidenced by the significant chi‐square value (Table 2). Degree of dominance estimations indicated that Black alleles are dominant over Brown alleles in this cross.

**Table 2:** Significance test values for epistasis and additive by sex, estimated degree of dominance by sex

	Male	Female
Epistasis test (*t* value)	15.64	127
Additivity test (*χ* ^2^)	0.44	4.00
Degree of dominance	−0.593	−0.010

Segregation variances ($\sigma_{S}^{2}$) determined using least squares were used to estimate factor number (*n*_e_). The estimated factor number was higher in females than males, indicating some factors may be X‐linked causing an overestimation in females and an underestimation in males (Otto & Jones, 2000). The square roots of the variances were used to estimate the number of effective factors for each sex. Finally, *n*_e_ was used to estimate a more unbiased number of factors (*n*) (Table 3). Again, differences in effective factor number and n are likely related to estimation biases based on sex.

**Table 3:** Estimated segregation variance by sex, estimated number of factors using the Castle–Wright estimator by sex, estimated number of large effect factor by sex and estimated number of factors using the Zeng adjustment by sex

	$\sigma_{S}^{2}$	*n*_e_	Large effect factors	*n*
Male	0.00372	10.20	8.10	29.17
Female	0.00681	12.87	6.99	43.21

A similar analysis of weight data, however, yielded a negative value for the Castle–Wright estimator. This was likely due to the strains not being differentiated enough for weight. This lack of divergence was expected since lines were partially selected based on similar size. This was done to reduce the number of potential factors affecting water consumption differences.

## 4 DISCUSSION

These results indicate many genes control the difference in water consumption between these two strains. In fact, this estimation is likely minimized due to violations of Castle–Wright estimator assumptions. To produce an unbiased prediction with the Castle–Wright estimator several assumptions must be met (Castle, 1921; Wright, 1968):

1. All alleles increasing the value of the phenotype are fixed in one line and all those that lower it are fixed in the other line.
2. Allelic effect differences are equal at all loci.
3. All loci are unlinked.
4. All alleles interact additively—no dominance or epistasis.

Expectedly, the data showed significant epistatic effects due to the quantitative nature of the trait (Cordell, 2002). Dominance effects were also observed which moved the *F*_1_ and *F*_2_ phenotypes nearer to the Black parent than the mid‐parent value. However, joint scale testing indicated the data were adequately fit by the additive model. This signalled the data could be analysed using the Castle–Wright estimator. It should be noted though, research (Huang & Mackay, 2016) has indicated that data, regardless of genetic architecture, can typically be fitted to the additive model. Previous comparisons of Castle–Wright estimators and quantitative trait loci (QTL) analyses have yielded similar results. This indicates the robustness of the estimator and reduces concerns about the additivity of the data (Wu, Bradshaw, & Stettler, 1997).

The number of genes controlling the difference in consumption may be surprisingly high from two closely related strains (Beck et al., 2000). However, previous work has indicated higher than expected genetic variance and divergence in the strains, particularly in regard to copy number variation (Cutler, Marshall, Chin, Baribault & Kassner, 2007). Further, the Brown strain has been noted for its high degree of genetic distinctiveness potentially related to mutation rate (Taylor, 1972) as well as a higher level of haplotypic introgression than typically observed in inbred strains (Yang et al., 2011). Regarding water consumption specifically, previous research has indicated differential androgen regulation in males (Melanitou, Cohn, Bardin, & Janne, 1987); however, no differences in androgen receptor or affinity (Kemp & Drinkwater, 1989). Brown females have also been noted for lowered ovarian hormone production (Maronpot, 2009) which can increase water intake (McGivern et al., 1996; Tarttelin & Gorski, 1971). This level of phenotypic diversity in the strain could explain the high levels of variance observed in the Brown strain.

## 5 CONCLUSION

In conclusion, the difference in water consumption between these two strains is controlled by many genes. This indicates a requirement for a very large number of animals to conduct a QTL analysis. It may be advisable to instead evaluate this trait using less genetically diverse strains such as C57BL/6J and C57BL/10J which still have a sufficient difference in water consumption (Tordoff et al., 2007).

## SOURCE OF FUNDING

Funding for this project provided by the University of Missouri Ag Experiment Station.

## CONFLICT OF INTEREST

The authors declare that they have no competing interests.

## ETHICAL STATEMENT

This study was designed and completed in accordance with University of Missouri ACUC Protocol #8656.

## References

[vms3192-bib-0001] Allan, J. A. (2001). The Middle East water question: Hydropolitics and the global economy. Choice Reviews Online, 39(04), 39–2423. 10.5860/CHOICE.39-2423

[vms3192-bib-0002] Bachmanov, A. , Reed, D. , Beauchamp, G. , & Tordoff, M. (2002). Food intake, water intake, and drinking spout side preference of 28 mouse strains. Behavior genetics, 32, 435–443. 12467341 10.1023/a:1020884312053 PMC1397713

[vms3192-bib-0003] Bachmanov, A. A. , Tordoff, M. G. , & Beauchamp, G. K. (1996). Ethanol consumption and taste preferences in C57BL/6ByJ and 129/J mice. Alcoholism: Clinical and Experimental Research, 20(2), 201–206. 10.1111/j.1530-0277.1996.tb01630.x PMC3638218 8730208

[vms3192-bib-0004] Barendse, W. (2017). Climate adaptation of tropical cattle. Annual Review of Animal Biosciences, 5(1), 133–150. 10.1146/annurev-animal-022516-022921 28199171

[vms3192-bib-0005] Beck, J. A. , Lloyd, S. , Hafezparast, M. , Lennon‐Pierce, M. , Eppig, J. T. , Festing, M. F. W. , & Fisher, E. M. C. (2000). Genealogies of mouse inbred strains. Nature Genetics, 24, 23 10.1038/71641 10615122

[vms3192-bib-0006] Castle, W. E. (1921). On a method of estimating the number of genetic factors concerned in cases of blending inheritance. Science. American Association for the Advancement of Science, 54, 93–96. 10.1126/science.54.1387.93 17793545

[vms3192-bib-0007] Cavalli, L. L. (1952). An analysis of linkage in quantitative inheritance. In *Quantitative inheritance. Papers read at a colloquium held at the Institute of Animal Genetics Edinburgh University under the auspices of the Agricultural Research Council April 4th to 6th, 1950* (pp. 135–144). HM Stationery Office. Retrieved from https://www.cabdirect.org/cabdirect/abstract/19541603514

[vms3192-bib-0008] Cockerham, C. C. (1986). Modifications in estimating the number of genes for a quantitative character. Genetics, 114(2), 659–664. Retrieved fromhttp://www.ncbi.nlm.nih.gov/pubmed/3770473. 3770473 10.1093/genetics/114.2.659 PMC1202962

[vms3192-bib-0009] Cordell, H. J. (2002). Epistasis: What it means, what it doesn't mean, and statistical methods to detect it in humans. Human Molecular Genetics, 11(20), 2463–2468. 10.1093/hmg/11.20.2463 12351582

[vms3192-bib-0010] Cutler, G. , Marshall, L. , Chin, N. , Baribault, H. , & Kassner, P. (2007). Significant gene content variation characterizes the genomes of inbred mouse strains. Genome research, 17, 1743–54. 10.1101/gr.6754607. 17989247 PMC2099583

[vms3192-bib-0011] de Fraiture, C. , & Wichelns, D. (2010). Satisfying future water demands for agriculture. Agricultural Water Management, 97(4), 502–511. 10.1016/j.agwat.2009.08.008

[vms3192-bib-0012] Falconer, D. S. , & Mackay, T. F. (1996). Introduction to quantitative genetics. Harlow, Essex: Longmans Green 10.1002/bimj.19620040211

[vms3192-bib-0013] Gale, J. G. , Mather, K. , & Jinks, J. L. (1977). Joint scaling tests. Heredity, 38(1), 47–51. 10.1038/hdy.1977.6 330469

[vms3192-bib-0014] Godfray, H. C. J. , Beddington, J. R. , Crute, I. R. , Haddad, L. , Muir, J. F. , Pretty, J. , … Toulmin, C. (2016). The challenge of feeding 9 billion people. Science, 327(5967), 812–818. 10.4337/9780857939388 20110467

[vms3192-bib-0015] Griffing, B. (1956). Concept of general and specific combining ability in relation to diallel crossing systems. Australian Journal of Biological Sciences, 9(4), 463 10.1071/BI9560463

[vms3192-bib-0016] Haag, M. T. , Wells, K. D. , & Lamberson, W. R. (2018). Genetic, maternal, and heterosis effects on voluntary water consumption in mice. Journal of Animal Science, 96(8), 3055–3063. 10.1093/jas/sky218 29860467 PMC6095341

[vms3192-bib-0017] Huang, W. , & Mackay, T. F. C. (2016). The genetic architecture of quantitative traits cannot be inferred from variance component analysis. PLoS Genetics, 12(11), e1006421 10.1371/journal.pgen.1006421 27812106 PMC5094750

[vms3192-bib-0018] Kemp, C. J. , & Drinkwater, N. R. (1989). Genetic variation in liver tumor susceptibility, plasma testosterone levels, and androgen receptor binding in six inbred strains of mice. Cancer Research, 49(18), 5044–5047. Retrieved fromhttp://www.ncbi.nlm.nih.gov/pubmed/2766275. 2766275

[vms3192-bib-0019] Lande, R. (1981). The minimum number of genes contributing to quantitative variation between and within populations. Genetics, 99(3–4), 541–553. Retrieved fromhttp://www.ncbi.nlm.nih.gov/pubmed/7343418. 7343418 10.1093/genetics/99.3-4.541 PMC1214520

[vms3192-bib-0020] Lynch, M. , & Walsh, B. (1998). Genetics and analysis of quantitative traits.Sunderland, MA: Sinauer 10.1086/318209

[vms3192-bib-0021] Maronpot, R. R. (2009). Biological basis of differential susceptibility to hepatocarcinogenesis among mouse strains. Journal of Toxicologic Pathology, 22(1), 11–33. 10.1293/tox.22.11 22271974 PMC3246016

[vms3192-bib-0023] Mather, K. , & Jinks, J. L. (1971). Biometrical genetics: The study of continuous variations. London, UK: Chapman and Hall.

[vms3192-bib-0024] Maupin, M. A. , Kenny, J. F. , Hutson, S. S. , Lovelace, J. K. , Barber, N. L. , & Linsey, K. S. (2010). Estimated use of water in the United States in 2010 circular 1405. US Geological Survey, 1405, 2–48. 10.3133/cir1405

[vms3192-bib-0025] McGivern, R. F. , Henschel, D. , Hutcheson, M. , & Pangburn, T. (1996). Sex difference in daily water consumption of rats: Effect of housing and hormones. Physiology and Behavior, 59(4–5), 653–658. 10.1016/0031-9384(95)02017-9 8778848

[vms3192-bib-0026] Melanitou, E. , Cohn, D. A. , Bardin, C. W. , & Janne, O. A. (1987). Genetic variation in androgen regulation of ornithine decarboxylase gene expression in inbred strains of mice. Molecular Endocrinology, 1(3), 266–273. Retrieved fromhttps://watermark.silverchair.com/mend0266.pdf?token=AQECAHi208BE49Ooan9kkhW_Ercy7Dm3ZL_9Cf3qfKAc485ysgAAAhowggIWBgkqhkiG9w0BBwagggIHMIICAwIBADCCAfwGCSqGSIb3DQEHATAeBglghkgBZQMEAS4wEQQMm7yPZ7BR_lhpWApJAgEQgIIBzeCQsLbINPV3KjQCUe1bN_U1k1CgokxarjgXxbvmgWWV66. 3453893 10.1210/mend-1-3-266

[vms3192-bib-0027] Otto, S. P. , & Jones, C. D. (2000). Detecting the undetected: Estimating the total number of loci underlying a quantitative trait. Genetics, 156(4), 2093–2107. 10.1534/genetics.104.029686 11102398 PMC1461347

[vms3192-bib-0028] Reed, D. R. , Bachmanov, A. A. , & Tordoff, M. G. (2007). Forty mouse strain survey of body composition. Physiology and Behavior, 91(5), 593–600. 10.1016/j.physbeh.2007.03.026 17493645 PMC2085171

[vms3192-bib-0029] Schlink, A. C. , Nguyen, M. L. , & Viljoen, G. J. (2010). Water requirements for livestock production: A global perspective. Revue Scientifique et Technique de l'OIE, 29(3), 603–619. 10.20506/rst.29.3.1999 21309458

[vms3192-bib-0030] Tarttelin, M. F. , & Gorski, R. A. (1971). Variations in food and water intake in the normal and acyclic female rat. Physiology and Behavior, 7(6), 847–852. 10.1016/0031-9384(71)90050-3 5167385

[vms3192-bib-0031] Taylor, B. A. (1972). Genetic relationships between inbred strains of mice. The Journal of Heredity, 63(2), 83–86. Retrieved from http://www.ncbi.nlm.nih.gov/pubmed/5031317 5031317 10.1093/oxfordjournals.jhered.a108235

[vms3192-bib-0032] Thaisz, J. , Tsaih, S.‐W. , Feng, M. , Philip, V. M. , Zhang, Y. , Yanas, L. , … DiPetrillo, K. (2012). Genetic analysis of albuminuria in collaborative cross and multiple mouse intercross populations. American Journal of Physiology‐Renal Physiology, 303(7), F972–F981. 10.1152/ajprenal.00690.2011 22859403 PMC3469684

[vms3192-bib-0033] Tordoff, M. , Bachmanov, A. , & Reed, D. (2007). Forty mouse strain survey of water and sodium intake. Physiology & Behavior, 91(5), 620–631. Retrieved fromhttp://www.sciencedirect.com/science/article/pii/S0031938407001187. 17490693 10.1016/j.physbeh.2007.03.025 PMC2085363

[vms3192-bib-0034] UN‐Water . (2013). Water Scarcity. *World Water Day 2013: International Year of Water Cooperation*, 2025. Retrieved from http://www.unwater.org/fileadmin/user_upload/watercooperation2013/doc/Factsheets/water_scarcity.pdf

[vms3192-bib-0035] Wright, S. (1968). The genetics of quantitative variability In Evolution and genetics of populations (Vol. 1, pp. 373–420). HM Stationery Office Retrieved from https://www.cabdirect.org/cabdirect/abstract/19541603507

[vms3192-bib-0036] Wu, R. , Bradshaw, H. D. , & Stettler, R. F. (1997). Molecular genetics of growth and development in Populus (Salicaceae). V. Mapping quantitative trait loci affecting leaf variation. American Journal of Botany, 84(2), 143–153. 10.2307/2446076 21712194

[vms3192-bib-0037] Yang, H. , Wang, J. R. , Didion, J. P. , Buus, R. J. , Bell, T. A. , & Welsh, C. E. , … De Villena, F. P. M. (2011). Subspecific origin and haplotype diversity in the laboratory mouse. Nature genetics, 43, 648–655. 10.1038/ng.847 21623374 PMC3125408

[vms3192-bib-0038] Zeng, Z. B. (1992). Correcting the bias of Wright's estimates of the number of genes affecting a quantitative character: A further improved method. Genetics, 131(4), 987–1001. Retrieved fromhttps://brcwebportal.cos.ncsu.edu/zeng/Genetics-92-Z.pdf. 1325390 10.1093/genetics/131.4.987 PMC1205108

